# Epidemiology of suicidal behavior in Catalonia, Spain

**DOI:** 10.1192/j.eurpsy.2025.333

**Published:** 2025-08-26

**Authors:** A. Giménez-Palomo, D. Hidalgo-Mazzei, E. Vieta, A. Benabarre

**Affiliations:** 1 Hospital Clínic of Barcelona, Barcelona, Spain

## Abstract

**Introduction:**

Suicide is a global public health issue. According to the World Health Organization (WHO), more than 700,000 people die by suicide worldwide each year. In 2019, it was the fourth leading cause of death among those aged 15 to 29 globally. In Spain, suicide has been the leading cause of external death in recent years, which has motivated in some regions the implementation of preventive strategies, such as *Codi Risc de Suïcidi* (Suicide Risk Code).

**Objectives:**

This study examines the epidemiology of suicidal behavior in the Catalan population between 2010 and 2019, exploring geographical disparities and the influence of different sociodemographic and clinical variables on the incidence of suicidal ideation and suicide attempt.

**Methods:**

All residents in Catalonia who attended the public health system from 2010 to 2019 were included in the study. Data were obtained from the *Program d’analítica de dades per a la recerca i la innovació en salut* (PADRIS) of the *Agència de Qualitat i Avaluació Sanitàries de Catalunya* (AQuAS). Data on geographical, sociodemographic, and clinical variables were collected for subsequent statistical analysis. Statistical significance was set at p<0.05.

**Results:**

A total of 1,421,510 individuals were included. Overall, 6921 cases of suicidal ideation (0.5%) and 1143 suicide attempts (0.1%) were registered, which an accumulated incidence of 487/100,000 inhabitants for the first outcome and 804/1,000,000 inhabitants for the second. From the whole sample, 83,592 individuals (5.9%) had a severe mental illness (SMI), whereas the proportion of patients with a SMI in the group of suicide attempters was 9.6%. The majority of individuals who attempted suicide were women (64.1%). The highest proportion of individuals with suicidal thoughts or attempts was found in the age range from 15 to 19 years. The presence of a somatic illness, low socioeconomic status, tobacco use and the presence of a severe mental illness were significantly associated with suicidal ideation and attempt. Those patients with suicidal behavior had a higher number of emergency visits during the 10-year period, and also a higher probability of being prescribed antidepressants, antipsychotics, benzodiazepines or lithium. Geographical disparities in the incidence of suicidal behavior were found, with specific regions showing higher rates. In a logistic regression analysis, all associations remained significant.

**Conclusions:**

This study highlights the association between sociodemographic and clinical factors, which should have been assessed in clinical practice, with suicidal behavior. Geographical disparities may be attributed to various factors, including access to mental health services and socioeconomic factors. This study underscores the importance of identifying specific profiles at a higher risk for committing suicide, and also to implement policies and prevention programs in order to address this public health issue comprehensively.

**Disclosure of Interest:**

None Declared

